# Optimising CN54gp140 plasmid delivery by comparing intramuscular and intradermal vaccination combinations with and without electroporation

**DOI:** 10.1186/1742-4690-9-S2-P316

**Published:** 2012-09-13

**Authors:** JF Mann, PF McKay, J Swales, K Klein, A Fiserova, A Cope, RJ Shattock

**Affiliations:** 1 Imperial College, London, UK

## Background

Prime boost vaccination studies employing plasmid DNA have been shown to significantly increase vaccine-induced immune responses with the added advantage of increasing vaccine-induced immune breadth. Furthermore, recent studies have shown that prevailing immune responses to DNA based vaccines can be substantially augmented by the immediate application of short pulses of electric current (electroporation) at the DNA injection site.

## Methods

We vaccinated mice with DNA via the intradermal (ID), intramuscular (IM) or combined (ID + IM) routes with and without electroporation (EP), using a Gene Transfer Unit (GTU®) plasmid expressing CN54gp140. Some mice were further boosted with recombinant trimeric CN54gp140. Female BALB/c mice (n=8) were vaccinated every three weeks. Serum and vaginal lavage samples were collected 1 week post each vaccination and tested by ELISA for anti-HIV-gp140 specific IgG and IgA. Spleen derived T cells were evaluated for antigen reactivity by IFN-g ELISpot using 2 peptide pools of 15mers overlapping by 11, spanning the full length of the gp140 molecule.

## Results

Antigen-specific T lymphocyte IFN-g cytokine expression and humoral antibody profiles were different after either ID or IM immunization or the combined route vaccinations. Moreover, we found that EP added an additional level of complexity by influencing T and B cell responses both quantitatively and qualitatively. As expected, these vaccinations did not elicit biologically significant levels of mucosal antigen-specific antibody responses.

## Conclusion

Multi-route vaccinations can enhance and subtly alter vaccine-induced B and T lymphocyte immunity. All of the vaccine routes benefited from enhancement to the DNA immunization using in vivo EP and the elicited immunity was again influenced by the enhanced transfection efficiency. These strategies are likely to be useful in the future design of vaccine modalities that seek to generate specific immune responses.

